# Osteogenic Differentiation of Renal Interstitial Fibroblasts Promoted by lncRNA *MALAT1* May Partially Contribute to Randall’s Plaque Formation

**DOI:** 10.3389/fcell.2020.596363

**Published:** 2021-01-11

**Authors:** Zewu Zhu, Fang Huang, Weiping Xia, Huimin Zeng, Meng Gao, Yongchao Li, Feng Zeng, Cheng He, Jinbo Chen, Zhiyong Chen, Yang Li, Yu Cui, Hequn Chen

**Affiliations:** Department of Urology, Xiangya Hospital, Central South University, Changsha, China

**Keywords:** Randall’s plaque, *MALAT1*, *Runx2*, osteogenic differentiation, renal interstitial fibroblasts

## Abstract

**Background:**

The current belief is that Randall’s plaques (RP) constitute a nidus for the formation of idiopathic calcium oxalate stones, but the upstream events in RP formation remain unclear. The present study aimed to investigate whether RP formation shares similarities with biomineralization and to illustrate the potential role played by the lncRNA *MALAT1* in osteogenic differentiation of human renal interstitial fibroblasts (hRIFs).

**Materials and Methods:**

Biomineralization and *MALAT1* expression were assessed in RP, and hRIFs were isolated and induced under osteogenic conditions for further experiments. The transcription initiation and termination sites in *MALAT1* were identified by 5′ and 3′ RACE. RNA immunoprecipitation assays and luciferase assays were used to validate the interactions among *MALAT1*, *Runx2* and miRNAs.

**Results:**

Upregulated expression of osteogenic markers and *MALAT1* was observed in RP and hRIFs induced with osteogenic medium. Biomineralization in RP and calcium phosphate (CaP) deposits in induced hRIFs were further verified by electron microscopy. Furthermore, overexpression of *MALAT1* promoted the osteogenic phenotype of hRIFs, while treatment with a miR-320a-5p mimic and knockdown of *Runx2* significantly suppressed the osteogenic phenotype. Further analysis showed that *MALAT1* functioned as a competing endogenous RNA to sponge miR-320a-5p, leading to upregulation of Runx2 and thus promoting osteogenic differentiation of hRIFs.

**Conclusion:**

Ectopic calcification and *MALAT1* partially contributed to the formation of RP, in which *MALAT1* might promote Runx2 expression to regulate osteogenic differentiation of hRIFs by sponging miRNA-320a-5p. The current study sheds new light on the lncRNA-directed mechanism of RP formation via a process driven by osteogenic-like cells.

## Introduction

Nephrolithiasis remains a global public health problem with increasing prevalence (Khan et al., 2016); calcium oxalate (CaOx) is the most common chemical component in renal calculi and is mostly idiopathic in nature (Canales and Hatch, 2014). Idiopathic CaOx stones are often attached to Randall’s plaques (RP), first identified in 1937 (Randall, 1937), which are regions of subepithelial mineralized tissue containing calcium phosphate (CaP) and are ultimately exposed at the surfaces of renal papillary tissue (Daudon et al., 2015; Evan et al., 2015a). RP are believed to originate in the renal tubular basement membrane of the loop of Henle; deposits migrate into the interstitium, where they develop into RP (Khan, 2015). Interestingly, the progression of CaP deposition in the renal interstitium to RP formation was found to be similar to pathological biomineralization (Evan et al., 2015b; Hsi et al., 2017), and previous studies advanced the hypothesis that the formation of RP involves a process driven by osteogenic-like cells (Gambaro et al., 2004; Khan and Canales, 2015). Despite the increasing number of studies and various resulting theories, the precise mechanisms of RP formation remain unclear (Wiener et al., 2018a). To further elucidate the molecular mechanisms of interstitial biomineralization in renal papillae, we focused on the osteogenic phenotype of human renal interstitial fibroblasts (hRIFs), since renal interstitial fibroblasts (RIFs) play an important role in the pathophysiology of renal interstitial diseases (Sharpe and Dockrell, 2012), and many fibroblasts have been proven to have the potential for osteoblast differentiation seen elsewhere, such as periodontal ligament fibroblasts (de Vries et al., 2018) and bronchial fibroblasts (Sabatini et al., 2005).

Long non-coding RNAs (lncRNAs), a class of non-protein-coding RNA transcripts of > 200 nucleotides (nt) in length, have been reported to regulate both the expression of various genes and biological processes through multiple mechanisms (Kapranov et al., 2007; Iyer et al., 2015). Metastasis-associated lung adenocarcinoma transcript 1 (*MALAT1*) is a highly conserved lncRNA located on chromosome 11q13 with a length of 8700 nt (Gutschner et al., 2013). Recently, accumulating evidence has verified that *MALAT1* plays a critical role in regulating osteogenic differentiation. *MALAT1* upregulates osterix expression to promote osteogenic differentiation in human bone marrow-derived mesenchymal stem cells by interacting with miRNA-143 (Gao et al., 2018); *MALAT1* is significantly upregulated in calcific valves and promotes osteoblastic differentiation of human aortic valve interstitial cells (Xiao et al., 2017). However, it remains unknown whether *MALAT1* plays a vital role in RP formation analogous to that in ectopic ossification, prompting us to investigate the potential functions of *MALAT1* in regulating osteogenic differentiation of hRIFs *in vitro*.

In the current study, we provided further evidence of the similarities between interstitial mineralization and ectopic calcification. Moreover, we demonstrated for the first time that hRIFs can form CaP deposits under osteogenic conditions and that *MALAT1*, a highly upregulated lncRNA in RP, promotes Runt-related transcription factor 2 (Runx2) expression to regulate osteogenic differentiation of hRIFs by sponging miRNA-320a-5p. Therefore, this study on the biological role of *MALAT1* in regulating the osteogenic phenotype of hRIFs may shed light on a new underlying molecular mechanism of RP formation and may even have important implications for future treatment or prevention of idiopathic CaOx renal stones.

## Materials and Methods

### Clinical Samples

This study was approved by the Xiangya Hospital Ethics Committee, and written informed consent was obtained from all participants before surgery. RP tissues were obtained with biopsy forceps (Karl Storz, Germany) from idiopathic CaOx stone formers during percutaneous nephrolithotomy, and normal renal tissues with papillae were obtained from patients with carcinoma of the upper urinary tract who underwent nephrectomy. A total of 32 RP tissue samples and 25 normal renal tissue samples were obtained in the Department of Urology, Xiangya Hospital, Central South University, between 2018 and 2019. The patients’ clinical characteristics are shown in Supplementary Table 1.

### Cell Culture and Identification

Human renal interstitial fibroblasts were isolated through mixed enzyme digestion combined with differential adhesion. Briefly, normal renal tissues without tumor invasion were obtained as previously described. Renal medulla tissue (10 g) was appropriately selected, finely minced with scissors, digested with 20 ml of enzyme mix [0.2 mg/ml Collagenase I (Sigma-Aldrich, United States), 0.2 mg/ml Collagenase IV (Sigma-Aldrich, United States) and 0.25 mg/ml trypsin (Sigma-Aldrich, United States)] and incubated at 37°C for 1 h with gentle agitation. The entire suspension was passed through a 100 μm cell strainer and centrifuged for 5 min at 800 × *g*. Cells were incubated in Dulbecco’s modified Eagle’s medium (BI, Israel) containing 10% fetal bovine serum (BI, Israel), 100 U/mL penicillin (BI, Israel), and 100 μg/mL streptomycin (BI, Israel) for 1 h at 37°C in the presence of 5% CO_2_, and the medium was then replaced with new medium to obtain adherent fibroblasts. For renal fibroblast identification, immunofluorescence staining was used to detect the expression of vimentin and E-cadherin.

Osteogenic differentiation of hRIFs was induced at approximately 70% confluence using osteogenic medium with 10 mM β-glycerophosphate (Sigma-Aldrich, United States), 200 μM ascorbic acid (Sigma-Aldrich, United States) and 100 nM dexamethasone (Sigma-Aldrich, United States), which was reported in a previous study (Huang et al., 2015). The medium was changed every 3 days.

### Cell Transfection

Recombinant lentiviruses carrying the full-length sequence of *MALAT1* (Len-*MALAT1*), short hairpin RNA targeting *MALAT1* (Len-sh-*MALAT1*), or short hairpin RNA targeting *Runx2* (Len-sh-*Runx2*), along with the corresponding scramble control lentiviruses (Len-Ctrl; Len-sh-Ctrl) were purchased from GenePharma (Shanghai, China). Supplementary Table 2 lists the sequences of the shRNAs, and Supplementary Table 3 lists the primer sequences used for plasmid construction to overexpress *MALAT1*. Viral infection of hRIFs was conducted at approximately 30% confluence. At 12 h post-infection, the medium was removed, the cells were washed with PBS, and fresh complete medium was added. The infected hRIFs were selected with 2 μg/ml puromycin for 5 days.

To modulate the expression of miR-320a-5p and miR-2114-5p in hRIFs, hRIFs at 70% confluence were transfected with a mimic (RiboBio, China) or inhibitor (RiboBio, China) of miR-320a-5p or miR-2114-5p using Lipofectamine 2000 (Invitrogen, United States) according to the manufacturer’s protocols. Additionally, Supplementary Table 4 shows the sequences of the mimics and inhibitors.

### Immunohistochemistry (IHC)

Tissues were fixed with 4% paraformaldehyde for 48 h, and 3-μm thick tissue sections were prepared. Sections were subsequently deparaffinized and rehydrated using conventional methods. Antigen retrieval was performed with Antigen Unmasking Solution (Vector Laboratories, United States) at 95°C for 10 min. Sections were immersed in 3% hydrogen peroxide in methanol for 15 min to quench endogenous peroxidase activity and were blocked with 10% normal goat serum for 30 min at room temperature. Sections were incubated with primary antibodies against Runx2 (ab76956; 1:200, Abcam, United Kingdom), Osteopontin (OPN; ab69498; 1:200, Abcam, United Kingdom), and osteocalcin (OCN; ab93876; 1:400, Abcam, United Kingdom) at 4°C overnight. Then, sections were incubated with the corresponding biotinylated secondary antibody (Vector Laboratories, United States) for 30 min at room temperature prior to reaction with DAB chromogen (Vector Laboratories, United States). Sections were counterstained with hematoxylin, and micrographs were acquired with a phase contrast light microscope (Leica, Germany).

### Immunofluorescence Staining

After treatment, hRIFs were fixed with 4% paraformaldehyde for 30 min and subsequently permeabilized in 0.1% Triton X-100 for 15 min. The cells were blocked with 10% goat serum at room temperature for 30 minutes prior to incubation with primary antibodies against vimentin (ab8978; 1:100; Abcam, United States) and E-cadherin (ab76055; 1:50; Abcam, United States) at 4°C for 12 h and were then treated with fluorophore-conjugated immunoglobulin G as the secondary antibody (1:150; Abcam) at room temperature for 1 h. DAPI (Sangon Biotechnology Co., Shanghai, China) was added at room temperature for 5 min. Images were acquired with a fluorescence microscope (Leica, Germany) and merged using Image-Pro Plus software (Media Cybernetics, Bethesda, MD, United States).

### RNA Fluorescence *in situ* Hybridization (RNA-FISH) Assay

Cy3-labeled sense and antisense probes were purchased from BoXin Company (Guangzhou, China). FISH assays were completed using a Fluorescence *in Situ* Hybridization Kit (BoXing, China) according to the instructions. Briefly, after cells were fixed and permeabilized using conventional methods, the cell layers were incubated with the probes in hybridization solution at 37°C overnight in the dark. Then, DAPI was added, and images were acquired with a fluorescence microscope (Leica, Germany).

### RNA Extraction and Quantitative Real-Time Polymerase Chain Reaction (qRT-PCR)

Total RNA was extracted using TRIzol reagent (Takara, Japan) and was then reverse transcribed into cDNA using a PrimeScript RT reagent Kit (Takara, Japan) or a miRNA reverse transcription PCR kit (Takara, Japan) as the reverse transcription kit. qRT-PCR was conducted using SYBR Green PCR reagent (Takara, Japan) in a real-time PCR system (Applied Biosystems, United States). The relative expression levels of RNAs were calculated by the 2^–ΔΔ*Ct*^ method. *U6* was selected as the internal control for microRNAs (miRs); GAPDH, for other RNAs. Additionally, PCR primers were synthesized by Sangon Biotech (Shanghai, China), and the sequence information is shown in Supplementary Table 5.

### 5′ and 3′ Rapid Amplification of cDNA Ends (RACE)

5′ and 3′ RACE were performed to identify the transcription initiation and termination sites in *MALAT1* with a SMARTer^TM^ RACE cDNA Amplification Kit (Clontech, CA, United States). Briefly, total RNA was isolated from RP tissues and hRIFs. RACE-ready cDNA was synthesized according to the manufacturer’s instructions. The obtained cDNA was purified on a 0.8% agarose gel, ligated to the linearized pRACE vector, and sequenced. The sequences of the designed gene-specific primers (GSPs) for the PCR step of the RACE procedure are listed in Supplementary Table 6. Additionally, the full-length sequence of *MALAT1* was further analyzed by PCR with fragment primers using a Prime Script^TM^ One Step RT-PCR Kit (Takara, Japan). The sequences of the fragment primers are listed in Supplementary Table 7.

### Western Blot Analysis (WB)

Total protein was isolated using RIPA lysis buffer (NCM, China) with 1% PMSF (NCM, China), and the concentration of protein was measured with a BCA Protein Assay Kit (Beyotime, China). The target proteins were separated from equal amounts of total protein by 12.5% sodium dodecyl sulfate-polyacrylamide gel electrophoresis (SDS-PAGE) and were then transferred to polyvinylidene fluoride (PVDF) membranes. After blocking non-specific binding with Quick Block solution (Beyotime, China), the membrane containing each target protein was incubated with specific primary antibodies at 4°C overnight. Additionally, the details of the primary antibodies are as follows: anti-GAPDH (ab8245; 1:4000, Abcam, United Kingdom); anti-Runx2 (ab76956; 1:2000, Abcam, United Kingdom); anti-Osteopontin (OPN; ab69498; 1:2000, Abcam, United Kingdom); anti-Osterix (ab209484; 1:3000, Abcam, United Kingdom); anti-osteocalcin (OCN; ab93876; 1:4000, Abcam, United Kingdom); and anti-Ago2 (cs204386; 1:2500, Millipore, United States). Horseradish peroxidase-conjugated goat anti-rabbit or goat anti-mouse IgG (1:5000; Proteintech, China) was used as the secondary antibody and incubated at room temperature for 1 h. Bands were visualized using an enhanced chemiluminescence (ECL) detection kit (NCM Biotech; China). GAPDH was used as the internal reference. The gray value of each band was measured with Quantity One software (Bio-Rad, Berkeley, CA, United States). The relative densities of the protein bands were calculated with ImageJ software.

### RNA Immunoprecipitation (RIP) Assay

The RIP assay was performed using a Magna RIP Kit (17-701; Millipore, United States) according to the manufacturer’s instructions. Briefly, hRIFs were lysed with RIP lysis buffer and were then incubated with RIP buffer containing protein A/G magnetic beads conjugated to an anti-Ago2 antibody (cs204386; Millipore, United States) or normal mouse immunoglobulin G (IgG; included in the kit: 17-701) as the negative control. WB was performed to test the efficiency of Ago2 immunoprecipitation in the incubated bead suspension. Then, immunoprecipitated RNA and total RNA (input control) extracted from whole-cell lysates were further analyzed by qRT-PCR.

### Luciferase Reporter Assay

To determine whether a miRNA directly targets *MALAT1* and the *Runx2* 3′-UTR, constructs containing the putative wild-type miRNA binding sites in *MALAT1* (*MALAT1*-wt) and the *Runx2* 3′-UTR (*Runx2*-wt) or mutant binding sites in *MALAT1* (*MALAT1*-mut) and the *Runx2* 3′-UTR (*Runx2*-mut) were cloned into the pmirGLO luciferase vector (Promega, United States). The constructed luciferase plasmids were transfected into hRIFs prior to transfection of the miR mimic or mimic negative control (NC-mimic) with Lipofectamine 2000 (Invitrogen, United States) according to the manufacturer’s protocol. After 48 h, Renilla and firefly luciferase activities were determined with a dual-luciferase reporter assay system (Promega, United States).

### Alizarin Red Staining (ARS)

After washing the cell layers with PBS, the cells were fixed with 4% paraformaldehyde for 30 min and washed 3 times with ddH2O. Then, the cell layers were stained with 1% Alizarin Red (Solarbio, China) for 10 min. After staining, the cells were washed gently with ddH_2_O 3 times, and the calcified nodules were visualized by staining as orange-red spots.

### ALP Activity Measurement

After removing the culture medium, the cell layers were washed with PBS, and lysis buffer was added (Beyotime, Shanghai, China). ALP activity was determined by the release of p-nitrophenol, as assessed by measuring the absorbance at 405 nm, using an ALP colorimetric assay kit (Beyotime, Shanghai, China).

### Transmission Electron Microscopy (TEM) and Scanning Electron Microscopy (SEM)

For TEM characterization of RP or normal renal papillary tissues, samples were immersed in 2.5% glutaraldehyde solution and postfixed in osmium tetroxide. After dehydration through a graded alcohol series, the sections of each side were examined by TEM (Tecnai G2 F20, FEI, United States). SEM was applied to examine the morphology and surface texture of the cells. Briefly, the cell layers were fixed with 4% paraformaldehyde for 30 min. After dehydration, the cell layers were coated with gold, scanning electron micrographs were acquired (Quanta-200, FEI, United States) with an accelerating voltage of 10 kV, and the chemical compositions were analyzed with energy-dispersive spectrometry (EDS).

### Bioinformatic Analysis

StarBase2.0^1^ and miRDB^2^ were used to predict the potential binding miRNAs of *MALAT1* or *Runx2*, and we selected miRNAs that putatively targeted both *MALAT1* and *Runx2* for analysis.

### Statistical Analysis

All experiments were repeated at least 3 times. Categorical variables were analyzed by chi-squared or Fisher’s exact tests, as appropriate. Continuous data are presented as the means ± standard deviation (SD) values. An independent samples t test was used to determine differences between two groups; one-way ANOVA was used to determine differences among three or more groups. The association of two variables was analyzed by two-tailed Pearson correlation analysis. A two-tailed P value of < 0.05 was considered statistically significant. Statistical analyses were performed using GraphPad Prism 8 software (GraphPad Software, La Jolla, CA, United States).

## Results

### Ossification-Like Calcification and Upregulated *MALAT1* Were Involved in RP Formation

**FIGURE 1:**
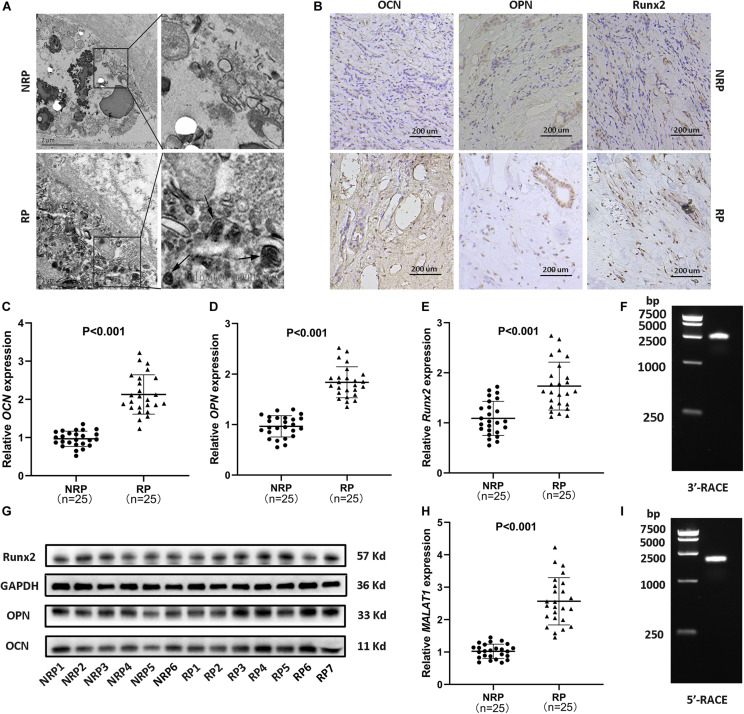
Randall’s plaques (RP) were associated with ectopic calcification and *MALAT1* upregulation. **(A)** Transmission electron microscopy (TEM) showed interstitial mineral crystals in RP, appearing as spheres (arrows) with alternating light and dark rings (30000 ×). **(B)** Osteogenic markers (OCN, OPN, Runx2) were detected by immunohistochemistry (IHC) in RP and normal renal papillae (NRP) (100 ×). **(C–E)** Relative mRNA expression levels of osteogenic markers were determined by qRT-PCR in RP (*n* = 25) and NRP (*n* = 25). **(F,I)** Agarose gel electrophoresis showed the PCR products from 5′ and 3′ RACE of *MALAT1* in RP tissues. **(G)** Protein expression levels of osteogenic markers were determined by WB in NRP (6/18) and RP (7/21). **(H)** Relative expression levels of *MALAT1* were determined by qRT-PCR in RP (*n* = 25) and NRP (*n* = 25). *GAPDH* was used as the internal control.

Calcium deposits, appearing as spheres with alternating light and dark rings (Figure 1A, arrows), were detected in RP by transmission electron microscopy (TEM). Subsequently, to assess the expression of osteogenic markers (Runx2, OCN, and OPN) in RP, immunohistochemical staining, qRT-PCR for RNA analysis, and WB were performed. The results consistently showed significantly increased expression of osteogenic markers in RP compared to normal renal papillae (NRP) (Figures 1B–E,G and Supplementary Figures 1d–f). Additionally, qRT-PCR showed that the relative expression of *MALAT1* was significantly upregulated in RP compared with NRP tissues (Figure 1H). Furthermore, linear regression analysis suggested that the mRNA expression levels of osteogenic markers were positively correlated with that of *MALAT1* (Supplementary Figures 1a–c). Therefore, we speculated that *MALAT1* might participate in a process driven by osteogenic-like cells to regulate RP formation.

The transcription initiation and termination sites of *MALAT1* in RP were successfully identified by 5′ and 3′ RACE (Figures 1F,I and Supplementary Figure 1g), and followed by sequencing (Supplementary Figures 1h,i). The full length was further confirmed by PCR with 5 pairs of fragment primers (Supplementary Figures 1g,j). The results showed that the transcript sequence was consistent with that of transcript variant 1 of *MALAT1* deposited in the NCBI database (NR_002819.4), and multiple studies have confirmed that *MALAT1* lacked open reading frames of consequential length and that no peptides were produced by translation of *MALAT1 in vitro* (Ji et al., 2003; Lin et al., 2007), suggesting that the *MALAT1* transcript in RP was a lncRNA.

### *MALAT1* Was Associated With Osteogenic Differentiation of hRIFs

**FIGURE 2:**
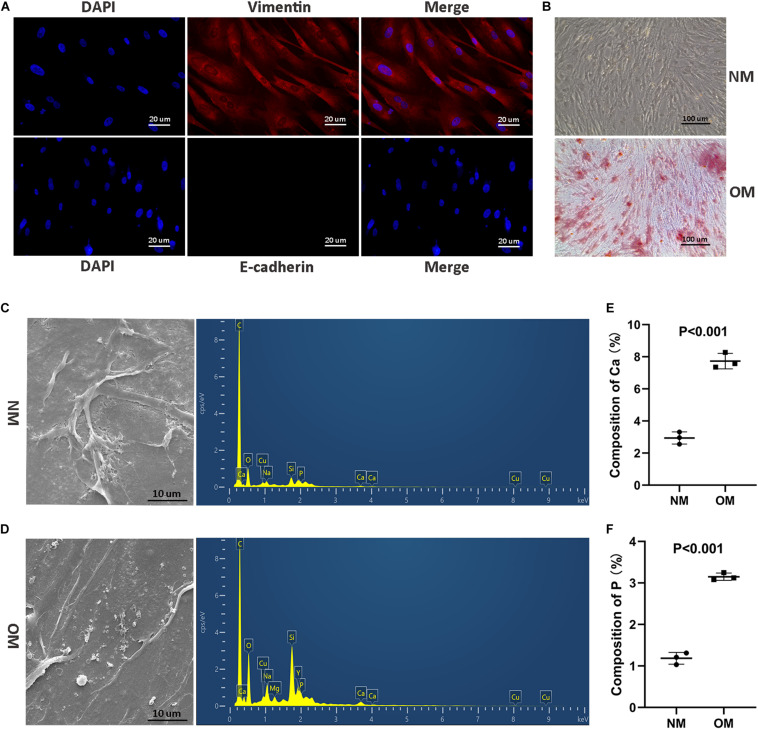
Identification of primary human renal interstitial fibroblasts (hRIFs) and osteogenic differentiation of hRIFs. **(A)** Fluorescence-labeled vimentin and E-cadherin proteins were visualized by fluorescence microscopy (200 ×) in isolated primary hRIFs. **(B)** Alizarin Red staining in hRIFs treated with osteogenic medium (OM) or normal medium (NM) for 14 days (100 ×). **(C)** Scanning electron microscopy (SEM) imaging and energy-dispersive spectrometry of hRIF layers cultured in NM for 14 days (5000 ×). **(D)** ESEM imaging and energy-dispersive spectrometry of hRIF layers cultured in OM for 14 days (5000 ×). **(E)** Elemental composition of Ca as determined by energy-dispersive spectrometry in hRIF layers cultured in NM and OM for 14 days. **(F)** Elemental composition of P as determined by energy-dispersive spectrometry in hRIF layers cultured in NM and OM for 14 days.

**FIGURE 3:**
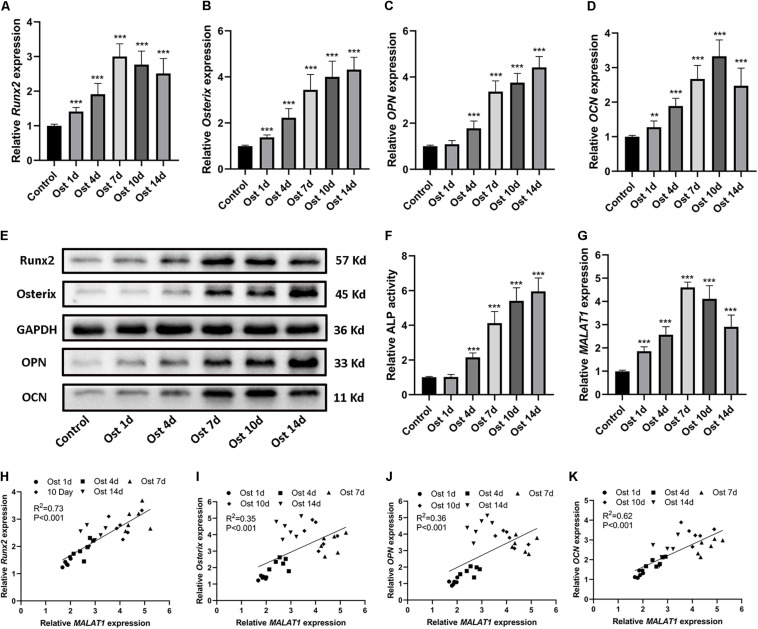
Upregulation of osteogenic markers and *MALAT1* in human renal interstitial fibroblasts (hRIFs) cultured under osteogenic conditions. **(A–D)** Relative mRNA expression levels of osteogenic markers (*Runx2, Osterix, OPN, OCN*) were determined by qRT-PCR 0, 1, 4, 7, 10 and 14 days after osteogenic induction. **(E)** Protein expression levels of osteogenic markers (Runx2, Osterix, OPN, OCN) were determined by WB 0, 1, 4, 7, 10 and 14 days after osteogenic induction. **(F)** The relative activity of alkaline phosphatase (ALP) was determined by the release of p-nitrophenol 0, 1, 4, 7, 10 and 14 days after osteogenic induction. **(G)** The relative expression level of *MALAT1* was determined by qRT-PCR 0, 1, 4, 7, 10 and 14 days after osteogenic induction. **(H–K)** Correlation analysis between *MALAT1* expression levels and the mRNA expression levels of osteogenic markers after osteogenic induction. *GAPDH* was used as the internal control. **P < 0.01; ***P < 0.001.

To investigate the osteogenic role hRIFs might play in RP formation, we isolated primary hRIFs from normal renal medulla tissues, and immunofluorescence staining showed that these hRIFs were positive for the marker vimentin and negative for E-cadherin (Figure 2A). After hRIFs were stimulated with osteogenic medium for 14 days, calcified nodule formation was detected by ARS (Figure 2B), and significantly increased levels of calcium (Ca) and phosphate (P) in the cell layers were identified by SEM and EDS (Figures 2C–F). Additionally, the expression levels of osteogenic markers (Runx2, Osterix, OPN and OCN; Figures 3A–E and Supplementary Figure 2a), ALP activity (Figure 3F) and the *MALAT1* expression level (Figure 3G) were greatly increased in an approximately induction time-dependent manner. Moreover, the results of linear regression analysis showed that *MALAT1* expression was positively associated with osteogenic marker expression (Figures 3H–K), which suggested a possible role for *MALAT1* in the regulation of osteogenic differentiation of hRIFs.

### *MALAT1* Promoted Osteogenic Differentiation of hRIFs

**FIGURE 4:**
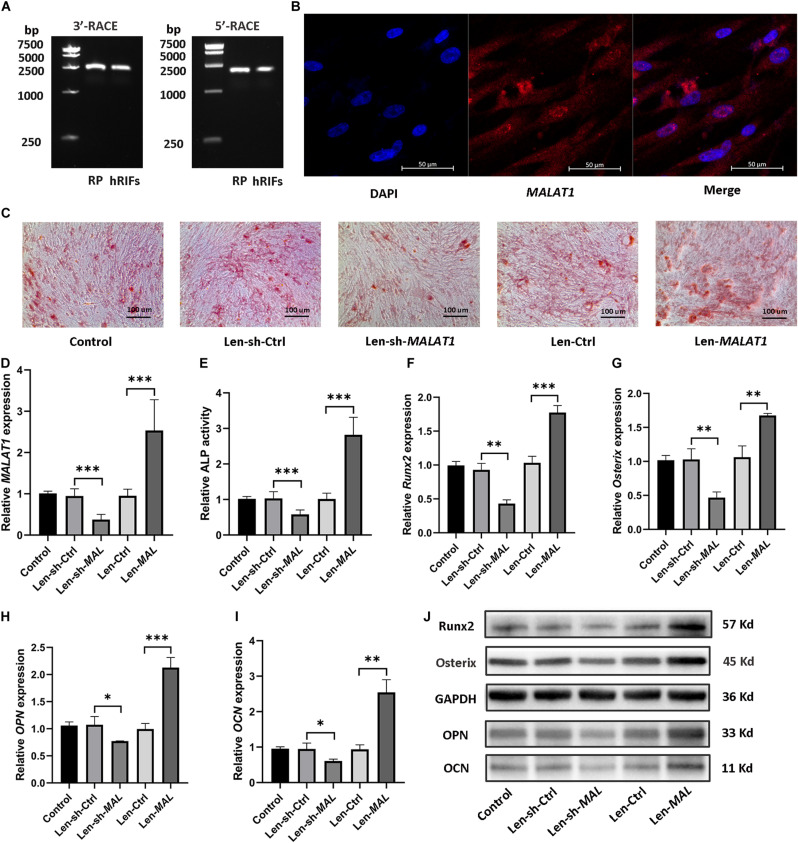
*MALAT1* promoted osteogenic differentiation of human renal interstitial fibroblasts (hRIFs). **(A)** Agarose gel electrophoresis showed the PCR products from the 5′ and 3′ RACE of *MALAT1* in hRIFs. **(B)** RNA fluorescence *in situ* hybridization (FISH) for *MALAT1* in isolated hRIFs. **(C)** Alizarin Red staining in transfected hRIFs 14 days after osteogenic induction (100 ×). **(D)** The relative *MALAT1* expression level was determined by qRT-PCR in transfected hRIFs 7 days after osteogenic induction. **(E)** Relative alkaline phosphatase (ALP) activity in transfected hRIFs 7 days after osteogenic induction. **(F–I)** Relative mRNA expression levels of osteogenic markers (*Runx2, Osterix, OPN, OCN*) were determined by qRT-PCR in transfected hRIFs 7 days after osteogenic induction. **(J)** Protein expression levels of osteogenic markers (Runx2, Osterix, OPN, OCN) were determined by WB in transfected hRIFs 7 days after osteogenic induction. *GAPDH* was used as the internal control. *P < 0.05; **P < 0.01; ***P < 0.001.

5′ and 3′ RACE of *MALAT1* in hRIFs showed results consistent with those in RP tissues (Figure 4A), and RNA-FISH showed that *MALAT1* was localized predominantly in the cytoplasm of hRIFs (Figure 4B). Subsequently, to examine whether the alteration in *MALAT1* could steer hRIFs toward an osteogenic phenotype, recombinant lentiviruses carrying small interfering RNA against *MALAT1* or carrying *MALAT1* were transfected into hRIFs, and *MALAT1* expression was confirmed by qRT-PCR (Figure 4D). The results showed that the knockdown of *MALAT1* decreased calcified nodule formation (Figure 4C) as well as ALP activity (Figure 4E) and reduced the expression levels of osteogenic markers (Figures 4F–J and Supplementary Figure 2b). In contrast, overexpression of *MALAT1* resulted in significantly increased calcified nodule formation (Figure 4C) and ALP activity (Figure 4E) and promoted the expression of osteogenic markers (Figures 4F–J and Supplementary Figure 2b). Taken together, these results indicated that *MALAT1* promoted osteogenic differentiation of hRIFs.

### *MALAT1* Directly Interacted With miR-320a-5p and miR-2114-5p

**FIGURE 5:**
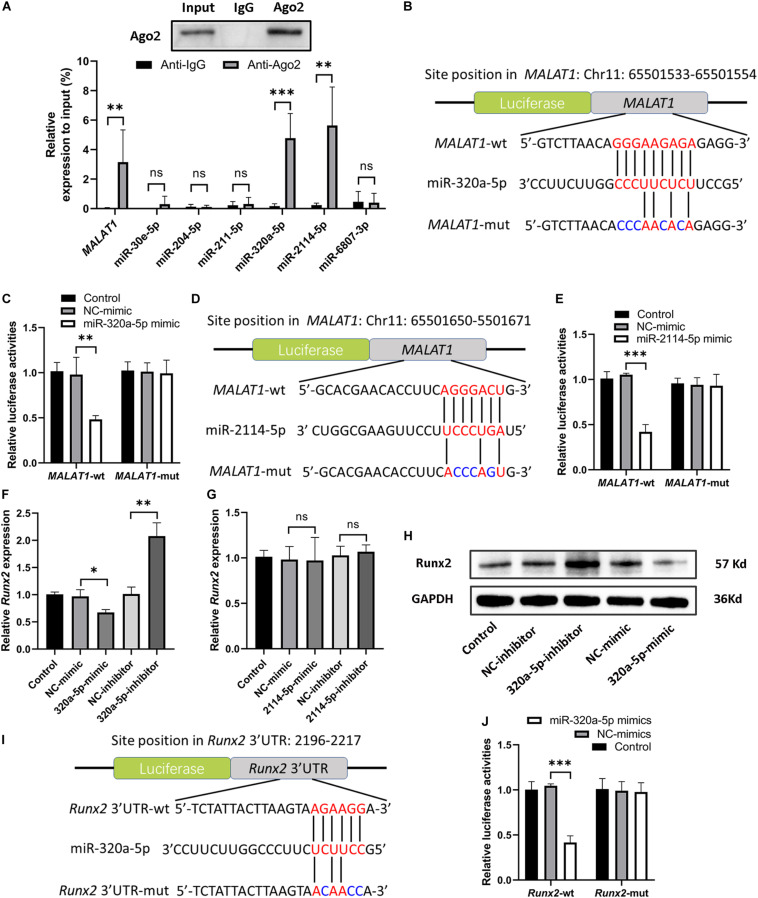
*MALAT1* bound to miR-320a-5p and miR-2114-5p, and miR-320a-5p directly interacted with *Runx2*. **(A)** Ago2 RIP (RNA immunoprecipitation) was performed, and qRT-PCR was used to determine the expression levels of *MALAT1* and predicted miRNAs relative to the input. **(B)** Schematic illustration of the predicted binding sequence of miR-320a-5p in wild-type *MALAT1* (*MALAT1*-wt) and the *MALAT1* mutant (*MALAT1*-mut). **(C)** Relative luciferase activity of *MALAT1*-wt and *MALAT1*-mut in human renal interstitial fibroblasts (hRIFs) treated with the miR-320a-5p mimic (320a-5p-mimic) or mimic negative control (NC-mimic). **(D)** Schematic illustration of the predicted binding sequence of miR-2114-5p in *MALAT1*-wt and *MALAT1*-mut. **(E)** Relative luciferase activity of *MALAT1*-wt and *MALAT1*-mut in hRIFs treated with the miR-2114-5p mimic (2114-5p-mimic) or NC-mimic. **(F)** The relative mRNA expression level of *Runx2* was determined by qRT-PCR in hRIFs treated with the miR-320a-5p mimic or miR-320a-5p inhibitor. **(G)** The relative mRNA expression level of *Runx2* was determined by qRT-PCR in hRIFs treated with the miR-2114-5p mimic or miR-2114-5p inhibitor. **(H)** The protein expression level of Runx2 was confirmed by WB in hRIFs treated with the miR-320a-5p mimic or miR-320a-5p inhibitor. **(I)** Schematic illustration of the predicted binding sequence of miR-320a-5p in the wild-type *Runx2*-3′UTR (*Runx2*-wt) and mutated *Runx2*-3’UTR (*Runx2*-mut). **(J)** Relative luciferase activity of *Runx2*-wt and *Runx2*-mut in hRIFs treated with the miR-320a-5p-mimic or NC-mimic. MicroRNA (miR) expression levels were normalized to those of *U6*; relative expression levels of other RNAs were normalized to those of *GAPDH*. *P < 0.05; **P < 0.01.

Recently, many studies have confirmed that cytoplasmic lncRNAs can sponge endogenous miRNAs at the posttranscriptional level to reduce the binding of miRNAs to their target osteogenesis-specific genes (Ju et al., 2019). Given that *MALAT1* was localized predominantly in the cytoplasm of hRIFs (Figure 4B) and that *MALAT1* expression displayed the strongest correlation with the expression of *Runx2* mRNA (*R*^2^ = 0.73; *P* < 0.001; Figure 3H) among osteogenic differentiation markers (Runx2, osterix (*R*^2^ = 0.35; Figure 3I), OPN (*R*^2^ = 0.36; Figure 3J), and OCN (*R*^2^ = 0.62; Figure 3K), we further investigated whether *MALAT1* regulates the expression of Runx2 by sponging miRNAs to promote osteoblastic differentiation of hRIFs. Bioinformatic analysis predicted that miR-30e-5p, miR-204-5p, miR-211-5p, miR-320a-5p, miR-2114-5p and miR-6807-3p can interact with both *MALAT1* and *Runx2*. Since lncRNAs and miRNAs were found to participate in the same RNA-induced silencing complex (RISC) through which miRNAs exert gene silencing effects (Gregory et al., 2005), an Ago2 RIP assay was performed to further verify the miRNAs that *MALAT1* can directly interact with. The results showed that *MALAT1*, miR-320a-5p and miR-2114-5p enrichment was significantly increased in Ago2 immunoprecipitates than in IgG immunoprecipitates (Figure 5A). Subsequently, we further investigated the expression of miR-320a-5p and miR-2114-5p in cells with knockdown of *MALAT1* or overexpression of *MALAT1*, and the results showed that the expression levels of both miR-320a-5p (Supplementary Figure 2c) and miR-2114-5p (Supplementary Figure 2d) were negatively correlated with the *MALAT1* expression level. Furthermore, a luciferase reporter assay was performed to investigate whether *MALAT1* directly interacts with miR-320a-5p (Figure 5B) or miR-2114-5p (Figure 5D), as predicted by the online bioinformatics database. The results demonstrated that the miR-320a-5p mimic significantly reduced the relative luciferase activity in hRIFs carrying *MALAT1*-wt, whereas no effect was observed in cells carrying *MALAT1*-mut (Figure 5C). Additionally, a similar result was found for miR-2114-5p in the luciferase reporter assay (Figure 5E). Collectively, our data suggested that *MALAT1* directly interacted with miR-320a-5p and miR-2114-5p.

### MiR-320a-5p Directly Interacted With *Runx2*

To investigate the effect of miR-320a-5p and miR-2114-5p on *Runx2* expression, hRIFs were transfected separately with a mimic or inhibitor of miR-320a-5p or miR-2114-5p, and the efficiency of the mimics and inhibitors was validated by qRT-PCR (Supplementary Figures 2e,f). The results showed that the miR-320a-5p mimic decreased the expression of Runx2; in contrast, the miR-320a-5p inhibitor significantly elevated the expression of Runx2 (Figures 5F,H and Supplementary Figure 3a). However, alteration of miR-2114-5p expression slightly influenced the expression of Runx2 (Figure 5G). Additionally, overexpression of miR-320a-5p suppressed the expression of osteogenic markers (OPN and OCN) and ALP activity, and the miR-320a-5p inhibitor led to the opposite effect (Supplementary Figures 3b–d). To further investigate whether miR-320a-5p directly interacts with the *Runx2*-3′-UTR, as predicted by bioinformatic analysis (Figure 5I), to inhibit osteoblastic differentiation of hRIFs, a dual-luciferase reporter assay was performed. The relative luciferase activity was significantly decreased when miR-320a-5p was cotransfected into cells carrying the wt *Runx2*-3′-UTR plasmid, whereas this effect was abolished by mutation of the predicted miR-320a-5p target site in the *Runx2* 3′-UTR (Figure 5J).

### *MALAT1* Sponged miR-320a-5p to Promote Osteoblastic Differentiation of hRIFs via Runx2 Upregulation

**FIGURE 6:**
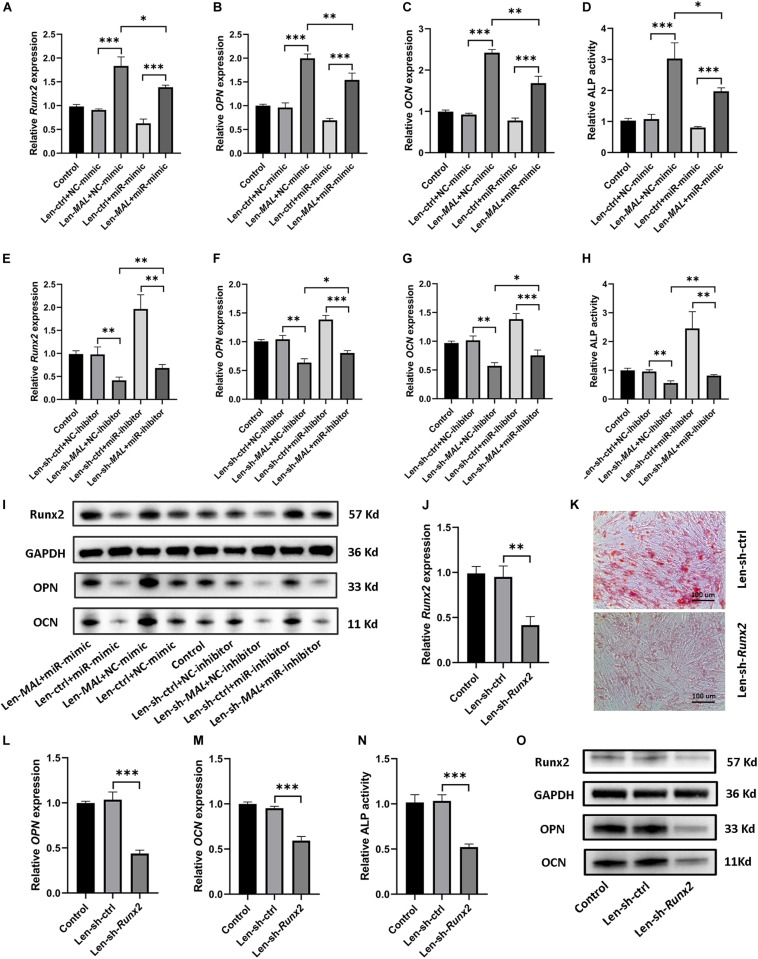
*MALAT1* sponged miR-320a-5p to promote osteogenic differentiation of human renal interstitial fibroblasts (hRIFs) via Runx2 upregulation. HRIFs were cotransfected with Len-sh-*MALAT1* or Len-sh-ctrl and the miR-320a-5p inhibitor or NC-inhibitor; **(A–C)** qRT-PCR was performed to determine the relative mRNA expression levels of osteogenic markers (*Runx2, OPN, OCN*), and **(D)** the release of p-nitrophenol was measured to determine the relative activity of alkaline phosphatase (ALP) 7 days after osteogenic induction. HRIFs were cotransfected with Len-*MALAT1* or Len-ctrl and the miR-320a-5p mimic or NC-mimic; **(E–G)** qRT-PCR was performed to determine the relative mRNA expression levels of osteogenic markers, and **(H)** the release of p-nitrophenol was measured to determine the relative activity of ALP 7 days after osteogenic induction. **(I)** Protein expression levels of osteogenic markers were determined by WB in cotransfected hRIFs 7 days after osteogenic induction. **(J)** qRT-PCR was used to determine the relative mRNA expression level of *Runx2* in hRIFs transfected with Len-sh-*Runx2* or Len-ctrl 7 days after osteogenic induction. **(K)** Alizarin Red staining in hRIFs transfected with Len-sh-*Runx2* or Len-ctrl 14 days after osteogenic induction (100 ×). **(L–N)** Relative mRNA expression levels of OPN and OCN and relative activity of ALP in hRIFs transfected with Len-sh-*Runx2* or Len-ctrl 7 days after osteogenic induction. **(O)** Protein expression levels of osteogenic markers were determined by WB in hRIFs transfected with Len-sh-*Runx2* or Len-ctrl 7 days after osteogenic induction. *GAPDH* was used as the internal control. *P < 0.05; **P < 0.01; ***P < 0.001.

**FIGURE 7:**
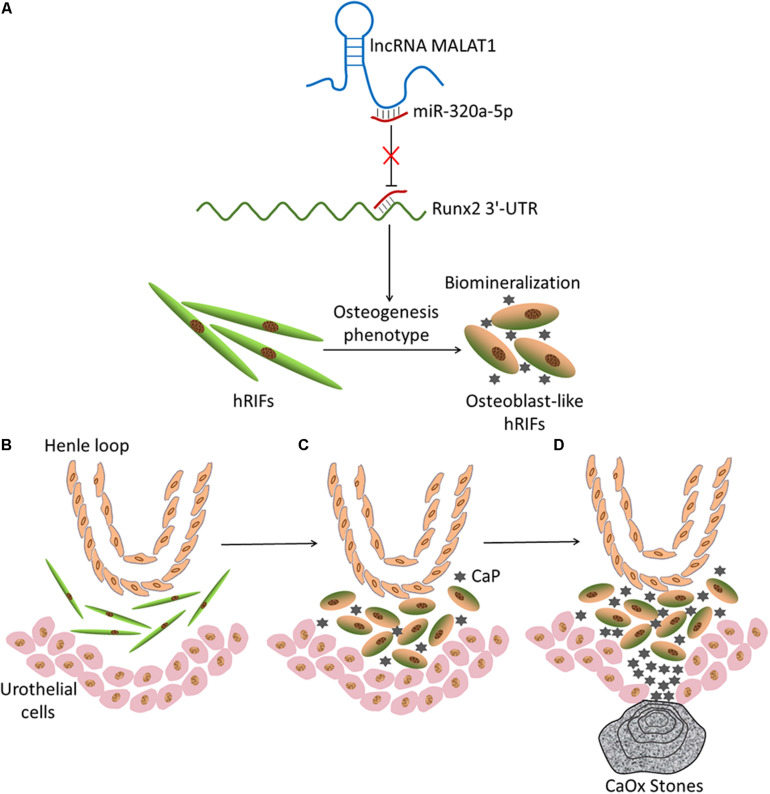
Schematic of the hypothetical theory of Randall’s plaque (RP) formation based on the current results. **(A)** Graphical abstract of the mechanism by which *MALAT1* promotes osteoblastic differentiation of hRIFs via a competing endogenous RNA (ceRNA) mechanism. **(B)** Normal condition of renal papillary tissue. Henle loop, human renal interstitial fibroblasts (hRIFs) and urothelial cells are illustrated. **(C)** Osteogenic differentiation of hRIFs and accumulation of calcium phosphate (CaP) deposits in the renal interstitium. **(D)** Subepithelial mineralized tissue with CaP becomes exposed at the surfaces of renal papillae, which eventually develop into a RP, and calcium oxalate (CaOx) is attached to the RP.

Given that *MALAT1* sequestered miR-320a-5p and that miR-320a-5p directly interacted with *Runx2*, we further examined whether *MALAT1* upregulated Runx2 by sponging miR-320a-5p. Our data revealed that overexpression of miR-320a-5p partially reversed the promotive effect of *MALAT1* on the expression of osteogenic differentiation markers (Runx2, OPN and OCN; Figures 6A–C,I and Supplementary Figure 3e) and ALP activity (Figure 6D). Additionally, *MALAT1* knockdown significantly decreased the expression levels of osteogenic differentiation markers, and cotransfection of the miR-320a-5p inhibitor partially rescued the expression of these markers, as expected (Figures 6E–I and Supplementary Figure 3g). Moreover, to determine whether *MALAT1* regulates osteogenic differentiation of hRIFs by modulating Runx2, lentiviruses carrying small interfering RNA against *Runx2* were generated to inhibit Runx2 expression (Figures 6J,O and Supplementary Figure 3f). The results showed that silencing *Runx2* significantly reduced calcified nodule formation, as determined by ARS (Figure 6K) and significantly decreased the expression of osteogenic differentiation markers (Figures 6L,M,O and Supplementary Figure 3f) and ALP activity (Figure 6N). Therefore, collectively, these results suggest that *MALAT1* inhibits miR-320a-5p to promote osteogenic differentiation of hRIFs via Runx2 upregulation (Figure 7).

## Discussion

With developments in endoscopic technology, it is widely accepted that RP constitute a nidus for the formation of idiopathic CaOx stones (Hsi et al., 2017). In recent decades, RP were found to contain CaP crystalline deposits and to be located in the tubular basement membranes and renal interstitium, appearing as spheres with alternating light and dark rings in which OPN was detected (Evan et al., 2005), and similarities between the context of RP formation and ectopic calcification were identified by light and electron microscopy techniques (Khan et al., 2012). Additionally, upregulation of osteogenesis-specific genes in the renal interstitium was identified in rats with hyperoxaluria induced by hydroxy-L-proline (Joshi et al., 2015). Moreover, in a recent study, nanoscale analysis of incipient RP was performed through electron energy loss spectrometry (Gay et al., 2020) and this study showed that some nanocalcifications exhibited similarities with physiological bone or cardiovascular pathological calcifications. In the current study, we similarly discovered laminated spherical crystals in RP by TEM and found that osteogenic markers were aberrantly upregulated in RP compared to normal renal papillary tissue. In combination with other published reports in this field (Kumar et al., 2006; Miyazawa et al., 2009; Mezzabotta et al., 2015), considerable evidence indicates that interstitial mineralization with CaP is akin to ectopic ossification (Gambaro et al., 2004; Wiener et al., 2018b). Although the current belief is that renal interstitial CaP deposition plays a vital role in the incipient formation of RP (Priante et al., 2017), the upstream events contributing to interstitial mineralization have not been thoroughly examined.

In the current study, we successfully isolated hRIFs and confirmed that hRIFs had the capacity to acquire an osteogenic phenotype under osteogenic conditions, as reported in our previous study (Zhu et al., 2020). Moreover, we performed SEM imaging and energy-dispersive spectrometry of hRIFs treated with OM. Interestingly, there were significantly increased levels of Ca and P in these cell layers compared to layers of hRIFs treated with normal medium, indicating that calcium phosphate precipitated to form calcified nodules, similar to hydroxyapatite, which was identified by electron diffraction and energy-dispersive X-ray microanalysis as the main component in crystal deposits in RP (Khan et al., 2012). Therefore, we have reason to assume that the biomineralization of hRIFs occurs in the upstream process of RP formation, and we further investigated the molecular mechanism underlying osteogenic differentiation of hRIFs.

Runx2 is well established as a master transcription factor that plays an essential role in osteoblastic differentiation of mesenchymal stem cells (Almalki and Agrawal, 2016). In the current study, we first investigated the role of Runx2 in osteogenic differentiation of hRIFs. Our data showed that downregulation of Runx2 decreased osteogenic differentiation marker expression and ALP activity and reduced calcified nodule formation in hRIFs, as determined by ARS, which verified the vital role played by Runx2 in regulating the osteogenic phenotype of hRIFs. Similarly, it was verified that Runx2 plays a core role in regulating osteogenic differentiation of other cells with osteogenic potential, such as adipose-derived mesenchymal stem cells (Yi et al., 2018) and human primary valve interstitial cells (Yu et al., 2018). The important role of Runx2 in osteogenic differentiation of multiple cell lineages suggests that the mechanism of Runx2 regulation may provide novel insight into renal interstitial mineralization and even RP formation, although universal evidence for Runx2 as one of the critical osteogenic regulators in RP formation is lacking (Jia et al., 2014; Evan et al., 2015b; Khan and Canales, 2015); this uncertainty may stem partially from the multiple steps involved in RP formation and the limited duration during which osteogenic signals are selectively activated (Khan and Gambaro, 2016).

Increasing evidence has verified that lncRNAs regulate osteoblast differentiation through transcription factor binding, chromatin modification, competing endogenous RNA mechanisms and other posttranscriptional mechanisms (Peng et al., 2018; Yang et al., 2018). The current study demonstrated that *MALAT1* expression was significantly elevated in RP compared with normal tissues. Intriguingly, the evidence generated in our study verified that *MALAT1* sponged miR-320a-5p to upregulate Runx2 expression and thus promoted the osteogenic differentiation of hRIFs. Similarly, other studies verified the identification of lncRNAs as competing endogenous RNAs (ceRNAs) that regulate Runx2 during osteogenic differentiation of other cells. *MALAT1* was found to promote Runx2-mediated osteogenic differentiation of adipose-derived mesenchymal stem cells by sponging miR-30 (Yi et al., 2018); in addition, knockdown of *TUG1* reduced Runx2 expression to inhibit osteogenic differentiation of human primary valve interstitial cells by interacting with miRNA-204-5p (Yu et al., 2018). Despite this evidence, it has been suggested that one lncRNA acting as a sponge could modulate multiple protein-coding genes (Xiao et al., 2017; Gao et al., 2018). *MALAT1* was reported to have a direct interaction with miR-142-3p and miR-129-5p (Liu et al., 2017), and both miRNAs were determined to have an important role in modulating the upstream regulators of *Runx2*, including beta-catenin (Hu et al., 2013) and signal transducer and activator of transcription 1 (STAT1) (Xiao et al., 2016). Thus, further investigations are needed to clarify whether *MALAT1* functions as a sponge to regulate the expression of other key regulators through interaction with different miRNAs. Moreover, lncRNAs participate in different regulatory mechanisms in the cytoplasm and nucleus (Ernst and Morton, 2013). Since *MALAT1* is distributed in the nucleus as well as the cytoplasm of hRIFs, further investigation of other functions of nuclear *MALAT1* and elucidation of its comprehensive role in promoting the osteogenic phenotype of hRIFs are needed.

In addition to osteogenic differentiation of hRIFs, osteoblast-like transdifferentiation of renal tubular epithelial cells (RTECs) may partially participate in the upstream events in RP formation. Previous studies showed that calcium ions promote the expression of osteogenic markers in primary RTECs and induce the differentiation of RTECs into cells with osteoblast-like phenotypes (He et al., 2015; Jia et al., 2015). Furthermore, it was verified that human renal proximal tubular cells (HK-2) can form CaP deposits after induction with osteogenic medium and that apoptosis might trigger the osteogenic phenotype of HK-2 cells during induction (Priante et al., 2019a). Since solutes are transported into the interstitium, and the interstitial papillary tip where RP form exhibits the highest concentration of solutes, including calcium ions (Wiener et al., 2018a), it is sensible to further clarify the potential interaction between calcium ions and hRIFs, and it will be interesting to identify whether there is cross-talk between hRIFs and RTECs, such as paracrine signaling, through which hRIFs and RTECs synergistically promote renal interstitial mineralization and even lead to RP formation.

Other molecular mechanisms, including immunity, oxidative stress, inflammation, and cell injury and/or death, may also participate in the formation of RP (Taguchi et al., 2017; Zhang et al., 2017; Priante et al., 2018; Chen et al., 2019). OPN was identified as the proinflammatory cytokine that contributed to calcium deposition in HK-2 cells treated with calcium oxymonohydrate and in a mouse model of renal calculi, and further study found that inhibition of the liver X receptor upregulated OPN to promote the initiation of nephrolithiasis (Chen et al., 2019). Genome-wide gene expression profiling was performed in RP (*n* = 23) and NRP (*n* = 7) (Taguchi et al., 2017), which revealed that the differentially expressed genes were enriched in the Akt/phosphatidylinositol 3-kinase, proinflammatory cytokine oxidative stress and proinflammatory cytokine pathways. Additionally, H19 was reported to promote renal tubular epithelial cell injury induced by calcium oxalate nephrocalcinosis through the HMGB1/11R4/NF-kappa B pathway (Liu et al., 2019), and GDNF (glial cell line-derived neurotrophic factor)-silenced HK-2 cells contributed to a calcification process via caspase-independent cell death (Priante et al., 2018; 2019a). Despite the advances in understanding the potential mechanism of RP formation, without effective pharmacotherapy, the prevalence and recurrence of nephrolithiasis remain high, leading to a substantial impact on both the affected individuals and healthcare systems (Morgan and Pearle, 2016). Thus, a better understanding of the pathogenetic mechanisms of RP is still required to guide the development of novel therapeutic and preventive interventions for CaOx stones.

To our knowledge, the current study is the first to identify that *MALAT1* is aberrantly overexpressed in RP and that knockdown of *MALAT1* greatly suppresses the osteogenic phenotype of hRIFs by targeting the miR-320a-5p/*Runx2* axis *in vitro*, indicating the critical role played by *MALAT1* in osteogenic differentiation of hRIFs through posttranscriptional mechanisms. However, we have to acknowledge several limitations of the current study. First, the unipapillary system in most animal models is inconsistent with the multipapillary system identified in humans, and there is no well-established animal model of RP formation, since a suitable animal model has not been thoroughly established to study renal interstitium mineralization with deposition of CaP (Morgan and Pearle, 2016). Thus, we did not verify whether *MALAT1* contributes to RP formation *in vivo*. Second, we only investigated the function of *MALAT1* in sponging miR-320a-5p, which targets *Runx2*, and it is necessary to explore whether *MALAT1* can function as a sponge to regulate the expression of other key regulators of the osteogenic phenotype in hRIFs. Third, *MALAT1* was expressed in both the nucleus and the cytoplasm of hRIFs. It should be noted that further study is necessary to explore the potential functions of nuclear *MALAT1*. Despite the limitations in the current study and the challenges in this field, this study provided novel insight into the role of the *MALAT1*/miR-320a-5p/*Runx2* signaling pathway in the potential molecular mechanism of RP formation, and further studies are anticipated to explore the roles of lncRNAs in the context of RP formation.

## Conclusion

Randall’s plaques formation shares similarity with biomineralization, in which osteogenic differentiation of hRIFs may play a critical role, and *MALAT1* can promote osteogenic differentiation of hRIFs by competitively binding to miR-320a-5p to upregulate Runx2. The current study provided novel mechanistic insight into a vital role for *MALAT1* as a miRNA sponge in regulating osteogenic differentiation of hRIFs, shedding new light on the lncRNA-directed mechanism of RP formation via a process driven by osteogenic-like cells.

## Data Availability Statement

The raw data supporting the conclusions of this article will be made available by the authors, without undue reservation.

## Ethics Statement

Approval was granted by the Ethics Committee of the Xiangya Hospital of Central South University (Proof Number: 201603035). Written informed consent to surgical procedures and for the publication of clinical data on the condition of anonymity was obtained preoperatively from all included patients.

## Author Contributions

ZZ and YC analyzed the data, wrote, and revised the manuscript. ZZ, FH, WX, HZ, MG, and YC performed the experiments. ZZ, FZ, CH, JC, ZC, YoL, and YaL collected the clinical samples. YC and HC conceived the project and revised the manuscript. All authors have approved the final version of the manuscript.

## Conflict of Interest

The authors declare that the research was conducted in the absence of any commercial or financial relationships that could be construed as a potential conflict of interest.

## Footnotes

1 http://starbase.sysu.edu.cn/index.php

2 http://mirdb.org/index.html
