# Supplementary material for: Osteogenic Differentiation of Renal Interstitial Fibroblasts Promoted by lncRNA MALAT1 May Partially Contribute to Randall’s Plaque Formation
Source: Front Cell Dev Biol. 2021 Jan 11;8:596363. doi: 10.3389/fcell.2020.596363 (PMC7829506; doi:10.3389/fcell.2020.596363)
Supplement: Supplementary file 1 [file Data_Sheet_1.docx]

**Supplementary Table 1.** The clinical characteristics of included patients.

| Parameters | Patients with CaOx stones (n=32) | Patients with renal tumor (n=25) | P value |
| --- | --- | --- | --- |
| Age (years), mean±SD | 54.2±11.6 | 52.4±7.4 | 0.516 |
| Gender (Male/Female) | 19/13 | 14/11 | 0.798 |
| BMI (kg/m^2^), mean±SD | 23.4±2.5 | 22.6±2.15 | 0.240 |
| Laterality (L/R), n | 15/17 | 14/11 | 0.494 |
| Degree of hydronephrosis*, n, % |  |  | 0.487 |
| None or mild | 25 (78.1%) | 22 (88.0%) |  |
| moderate | 7 (21.9%) | 3 (12.0%) |  |
| Comorbidities, n (%) |  |  | 0.729 |
| Diabetes mellitus | 5 (15.6%) | 4 (16.0%) |  |
| Hypertension | 9 (28.1%) | 5 (20.0%) |  |
| Renal insufficiency | 3 (9.4%) | 1 (4.0%) |  |
| Stone burden (mm^2^)**, mean±SD | 947±393 |  | - |
| Tumor size (cm), mean±SD | - | 6.2±1.5 | - |

*The hydronephrosis degree was evaluated according to the Society of Fetal Urology grading system. Patients with severe hydronephrosis were excluded. **Stone burden was calculated by the formula (0.785×length_max_×width_max_) according to CROES; if multiple stones were noted, the total burden was calculated by summing the burden of all the stones. BMI=Body Mass Index; CaOx=calcium oxalate; SD=standard deviation.

**Supplementary Table 2.** ShRNA sequences designed for *MALAT1* and *Runx2*

| Gene | shRNA sequences |
| --- | --- |
| sh-*MALAT1* | Sense: 5’-CCGGGGTTGAGATGAAGCTTCTTCTCGAGAAGAAGCTTCATCTCAACCTTTTTG-3’ |
|  | Anti-sense: 5’-AATTCAAAAAGGTTGAGATGAAGCTTCTTCTCGAGAAGAAGCTTCATCTCAACC-3’ |
| sh-NC for *MALAT1* | Sense: 5’-CCGGTTCTCCGAACGTGTCACGTTTCAAGAGAACGTGACACGTTCGGAGAATTTTTG-3’ |
|  | Anti-sense: 5’-AATTCAAAAATTCTCCGAACGTGTCACGTTCTCTTGAAACGTGACACGTTCGGAGAA-3’ |
| sh-*Runx2* | Sense: 5’-CCGGGCTACCTATCACAGAGCAATTCTCGAGAATTGCTCTGTGATAGGTAGCTTTTTG-3’ |
|  | Anti-sense: 5’-GATCCAAAAAGCTACCTATCACAGAGCAATTCTCGAGAATTGCTCTGTGATAGGTAGC-3’ |
| sh-NC for *Runx2* | Sense: 5’-CCGGTTCTCCGAACGTGTCACGTTTCAAGAGAACGTGACACGTTCGGAGAATTTTTG-3’ |
|  | Anti-sense: 5’-AATTCAAAAATTCTCCGAACGTGTCACGTTCTCTTGAAACGTGACACGTTCGGAGAA-3’ |

**Supplementary Table 3.** Primer sequences for plasmid construction to overexpression of *MALAT1*

| Gene | Forward primer 5′–3′ | Reverse primer 5′–3′ |
| --- | --- | --- |
| *MALAT1* | CCCAAGCTTGTAAAGGACTG | CCGGAATTCAACGGGTCATCA |

**Supplementary Table 4.** Mimics and inhibitor sequences for miRNAs

| Gene | Sequences 5′–3′ |
| --- | --- |
| MiR-320a-5p-mimics | GCCUUCUCUUCCCGGUUCUUCC |
| MiR-2114-5p-mimics | UAGUCCCUUCCUUGAAGCGGUC |
| MiR-320a-5p-inhibitor | GGAAGAACCGGGAAGAGAAGGC |
| MiR-2114-5p-inhibitor | GACCGCUUCAAGGAAGGGACUA |
| NC-mimics | Involved in the kit |
| NC-inhibitor | Involved in the kit |

**Supplementary Table 5.** Primer sequences for qRT-PCR

| Gene | Forward primer 5′–3′ | Reverse primer 5′–3′ |
| --- | --- | --- |
| *MALAT1* | AAAGCAAGGTCTCCCCACAAG | GGTCTGTGCTAGATCAAAAGGCA |
| *Runx2* | CACCATGTCAGCAAAACTTCTT | TCACGTCGCTCATTTTGC |
| *Osterix (SP7)* | GACTGCAGAGCAGGTTCCTC | TAACCTGATGGGGTCATGGT |
| *Osteocalcin (OCN)* | CTTTGTGTCCAAGCAGGA | CTGAAAGCCGATGTGGTCAG |
| *Osteopontin (OPN)* | GAAGTTTCGCAGACCTGACAT | GTATGCACCATTCAACTCCTCG |
| *GAPDH* | AACGTGTCAGTGGTGGACCTG | AGTGGGTGTCGCTGTTGAAGT |
| miR-320a-5p | CGTTCCCTTTGTCATCCTATGCCT | Involved in the kit |
| miR-2114-5p | TAGTCCCTTCCTTGAAGCGGTC | Involved in the kit |
| *U6* | CTCGCTTCGGCAGCACA | AACGCTTCACGAATTTGCGT |

**Supplementary Table 6.** Gene-specific primers (GSP) for 5′ and 3′-rapid amplification of cDNA ends (RACE) analysis

|  | GSP (outer) 5′–3′ | GSP (inner) 5′–3′ |
| --- | --- | --- |
| 5’-RACE | ATACTTCTGCACCACCAGAAATT | ATGCGTTAACTAGGCTTTAAATGACGC (1842 bp) |
| 3’-RACE | ATCTCTGAAGGCTCTATGAAAGGAATA | TCTTTAATGG ACCAGATCAG GATTTGA (2768 bp) |

**Supplementary Table 7.** Fragmented primers (FP) for verifying the full length of *MALAT1* by PCR amplification.

| Fragmented primers (FP) | Forward primer 5′–3′ | Reverse primer 5′–3′ |
| --- | --- | --- |
| FP 1 (795bp) | GAGATTAAACCGAAGGTGATTA | ACTTCCGTTACGAAAGTCCTTCAC |
| FP 2 (1291bp) | GGTGTTTACGTAGACCAGAACCA | TAAACTGTAAACCTGTGGTGGTCTG |
| FP 3 (862bp) | ACTTCCTCACCCTGAATTCGTT | GTTACTTGCCAACTTGGAAGTTGA |
| FP 4 (1049bp) | CAGAAGAGCTTGAGTAGGCCA | GCAGATAATGTTCTCATCAGTAG |
| FP 5 (1439bp) | CAGCTCCTTGGTGAATTGATAAG | GGATGAAATGCCTCTGCAAAGG |
