# Supplementary material for: Osteogenic Differentiation of Renal Interstitial Fibroblasts Promoted by lncRNA MALAT1 May Partially Contribute to Randall’s Plaque Formation
Source: Front Cell Dev Biol. 2021 Jan 11;8:596363. doi: 10.3389/fcell.2020.596363 (PMC7829506; doi:10.3389/fcell.2020.596363)
Supplement: Supplementary file 2 [file Data_Sheet_2.docx]

### Supplementary Figures

**
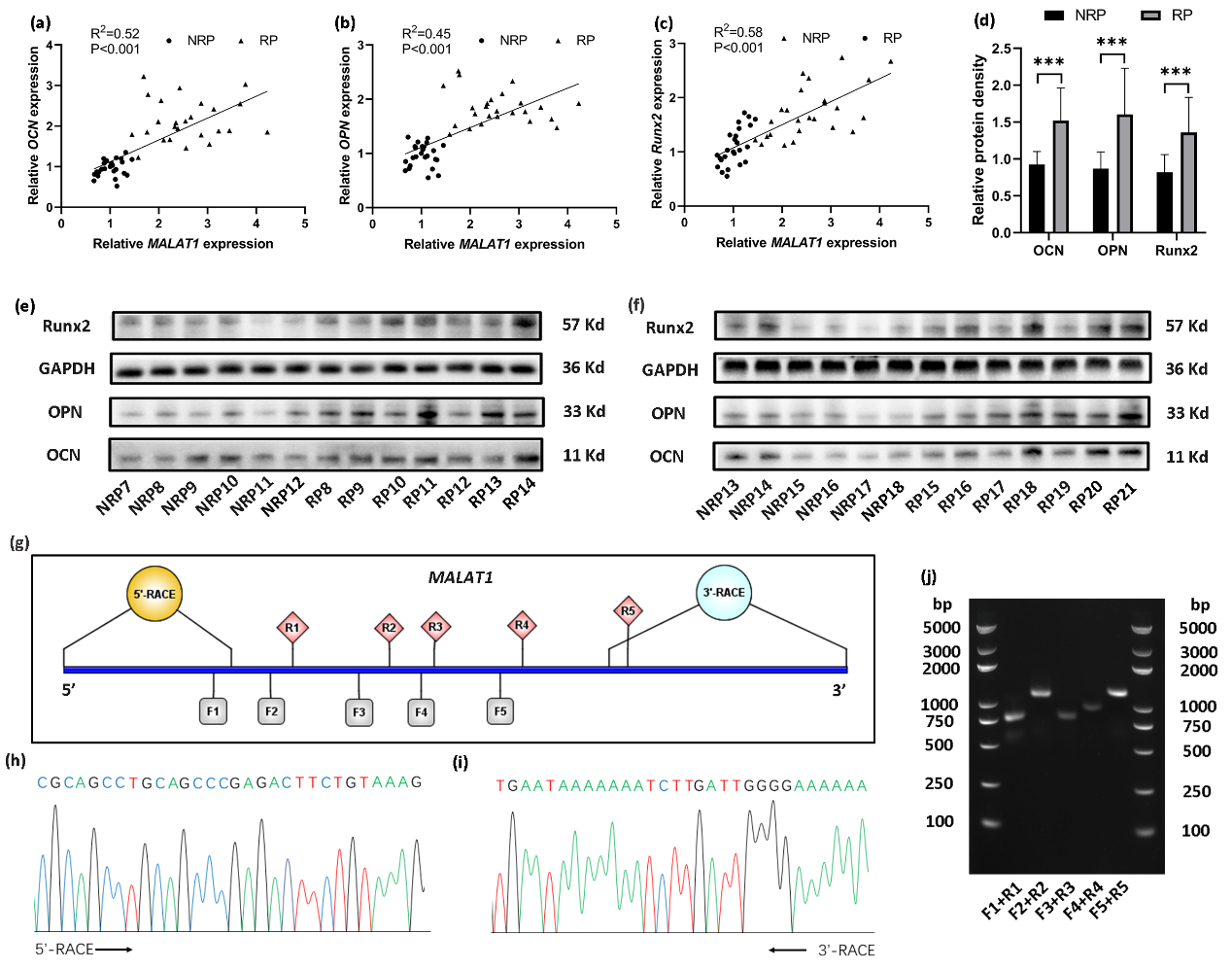
**

**Supplementary Fig. 1** **(a-c)** Correlation analysis between the *MALAT1* expression level and the mRNA expression levels of osteogenic markers (*OCN, OPN and Runx2*) in Randall’s plaques (RP) and normal renal papillae (NRP). **(d)** ImageJ software was used to analyze the relative densities of the protein bands of osteogenic markers detected by WB in RP (n=18) and NRP (n=21). **(e-f)** Protein expression levels of osteogenic markers were determined by WB in NRP (12/18) and RP (14/21). **(g)** Schematic of 5′ and 3′ RACE (rapid amplification of cDNA ends) and PCR amplification with 5 pairs of fragment primers to determine the full-length sequence of *MALAT1*. **(h-i)** Sequencing of RACE products to annotate the boundaries between the universal anchor primers and *MALAT1* sequences. **(j)** Agarose gel electrophoresis showed the PCR products amplified by 5 pairs of fragment primers to further confirm the full-length *MALAT1*. *GAPDH* was used as the internal control. ***P<0.001.

**
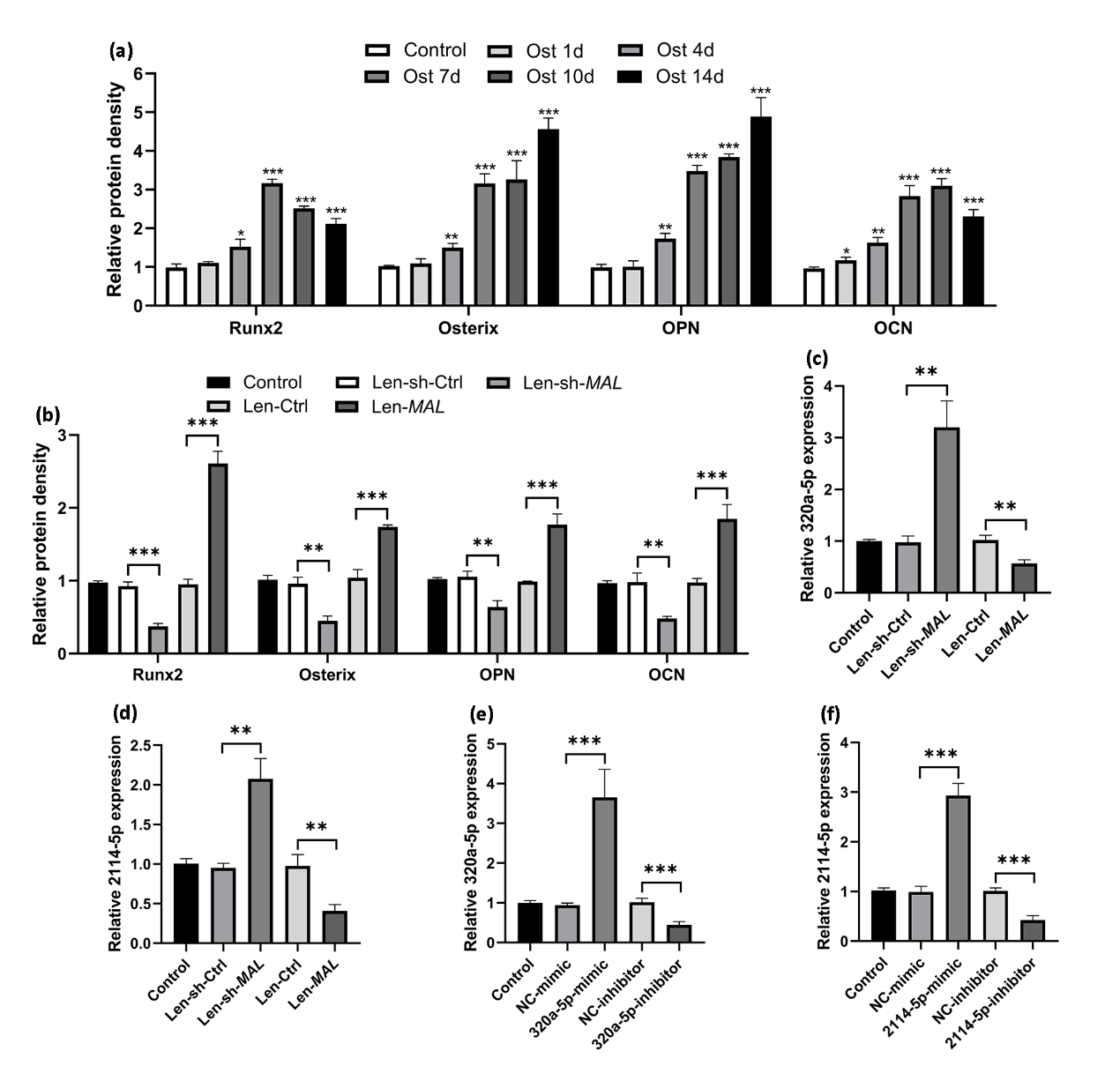
Supplementary Fig. 2 (a)** ImageJ software was used to analyze the relative densities of the protein bands of osteogenic markers detected by WB 0, 1, 4, 7, 10 and 14 days after osteogenic induction of human renal interstitial fibroblasts (hRIFs). **(b)** ImageJ software was used to analyze the relative densities of the protein bands of osteogenic markers detected by WB in hRIFs transfected with Len-sh-*MALAT1* or Len-*MALAT1* 7 days after osteogenic induction. **(c)** The relative expression level of miR-320a-5p was determined by qRT-PCR in hRIFs transfected with Len-sh-*MALAT1* or Len-*MALAT1* 7 days after osteogenic induction. **(d)** The relative expression level of miR-2114-5p was detected by qRT-PCR in hRIFs transfected with Len-sh-*MALAT1* or Len-*MALAT1* 7 days after osteogenic induction. **(e)** qRT-PCR was used to determine the relative expression level of miR-320a-5p in hRIFs transfected with the miR-320a-5p mimic or inhibitor. **(f)** qRT-PCR was used to determine the relative expression level of miR-2114-5p in hRIFs transfected with the miR-2114-5p mimic or inhibitor. *U6* was selected as the internal control for microRNAs (miRs); *GAPDH* was used for others. *P <0.05; **P <0.01; ***P<0.001.

**
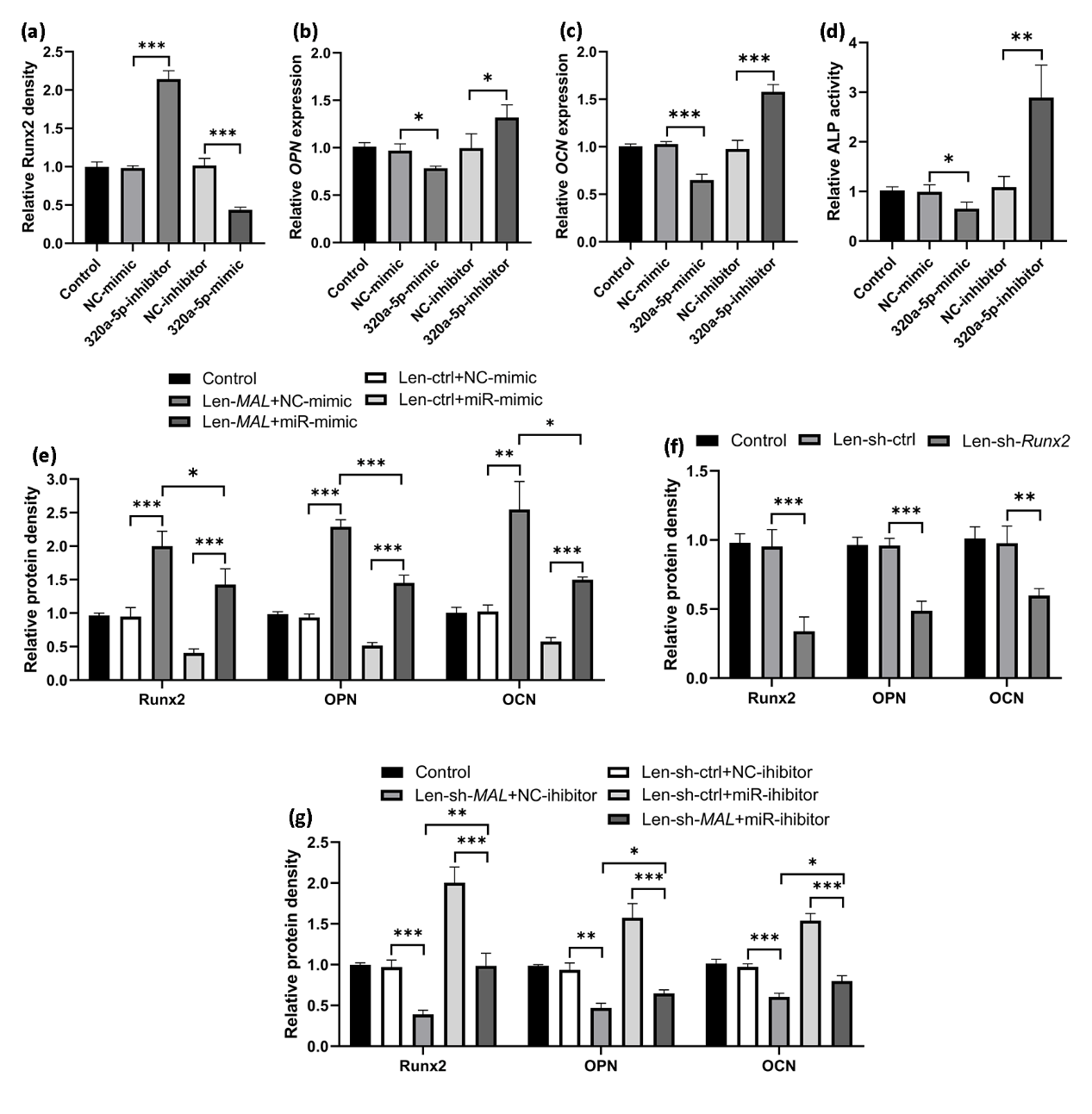
Supplementary Fig. 3 (a)** ImageJ software was used to analyze the relative densities of the protein bands of Runx2 detected by WB in hRIFs treated with the miR-320a-5p mimic or miR-320a-5p inhibitor 7 days after osteogenic induction. **(b-c)** Relative expression levels of *OPN* and *OCN* were determined by qRT-PCR in hRIFs transfected with the miR-320a-5p mimic or miR-320a-5p inhibitor 7 days after osteogenic induction. **(d)** Relative activity of alkaline phosphatase (ALP) in hRIFs transfected with the miR-320a-5p mimic or miR-320a-5p inhibitor 7 days after osteogenic induction. **(e)** HRIFs were cotransfected with Len-sh-*MALAT1* or Len-sh-ctrl and the miR-320a-5p inhibitor or NC-inhibitor and subjected to osteogenic induction for 7 days. ImageJ software was used to analyze the relative densities of the protein bands of osteogenic markers detected by WB. **(f)** ImageJ software was used to analyze the relative densities of the protein bands of osteogenic markers detected by WB in hRIFs transfected with Len-sh-*Runx2* or Len-ctrl 7 days after osteogenic induction. **(g)** HRIFs were cotransfected with Len-*MALAT1* or Len-ctrl and the miR-320a-5p mimic or NC-mimic and subjected to osteogenic induction for 7 days. ImageJ software was used to analyze the relative densities of the protein bands of osteogenic markers detected by WB. *GAPDH* was used as the internal control. *P <0.05; **P <0.01; ***P<0.001.
